# Abstracts from Foreign Journals

**Published:** 1903-02

**Authors:** S. J. J. Harger

**Affiliations:** Philadelphia, Pa.


### ABSTRACTS FROM FOREIGN JOURNALS.

### FRENCH.

Under the Direction of S. J. J. Harger, V.M.D.,
PHILADELPHIA, PA.

Atrophy of the Optic Papilla. Atrophy of the optic papilla with blindness may result from various traumatisms to the head. In Remond's case it was associated with the lesions of disseminated choroiditis.

The mare under the saddle had shown evidence of imperfect vision, and when the left eye was blindfolded was apparently blind

*Examination of the Eye.* Volume and tension normal; pupil contracted, but dilating completely after the use of atropine; media normal; papilla very pale, anæmic, with a clear and regular outline; papillary and retinal vessels indistinct and small. The morbid paleness of the papilla was very evident by comparing it with that of the left eye. Above the optic papilla there were numerous areas of disseminated choroiditis, with a whitish, grayish, or reddish base. A subsequent examination showed that the optic papilla was absolutely without vessels, pigmented, and deformed at its inferior border by the progressive choroiditis.

According to some writers, disseminated choroiditis is a benign affection, but only on condition that it remains confined to this part of the uveal tract; on the other hand, the inflammation is liable to spread to the optic papilla, and thus cause its atrophy.

The treatment, which was ineffective, consisted of 20 centigrammes of nitrate of pilocarpine every ten days and 20 grammes of iodide of potash daily.—*Rec. de Med. Vet.*

Arrhenal. Gautier has discovered a number of arsenico-methylic products, among which is arrhenal. Thébault (*Bulletin de Therap.*) published the following conclusions:

1. Arrhenal has given brilliant results in all cases in which arsenic is indicated, and in much less time than is required for the cacodylates or Fowler's solution.

2. Excepting some accidents, which may have resulted from too large doses of an impure drug, no toxic effects have been observed.

Arrhenal possesses a great advantage over the arsenical preparations that possess therapeutic value.—*Rec. de Med. Vet.*

Acute Ulcerative Endocarditis Complicated with Infarcts. Subject, a bitch, succumbing in three days to the removal of a tumor of the mammary gland.

At the necropsy Petit found the following alterations: Acute generalized enteritis, congestion of the liver, and infarcts in the spleen and kidneys. The heart showed an ulcerative endocarditis, localized especially upon the aortic sigmoid valves. Two of these valves were the seat of a large ulceration and covered by a loosely adherent layer of fibrinous deposit, which was the probable origin of the infarcts. The third valve had a perforation in the centre covered by a layer of fibrin. This lesion of the aortic valves caused aortic insufficiency, which was not recognized during life.

The operation possibly hastened the evolution of the symptoms, but the cause of the previous existence of the endocarditis was not determined.—*Rec. de Med. Vet.*

Rectal Insufflation of Air in Certain Forms of Colic. Since air forced into a collapsed tortuous tube will cause the latter to assume its normal form, Hermans conceived the application of this same principle in abdominal disorders due to a changed position of the intestines.

One case was in this manner treated with success. The subject, a foal, two-and-one-half months old, had suffered from colic for thirty-six hours, which had resisted the use of saline purgatives and repeated clysters. The animal was lying down, extremely depressed, in a state of profound coma, and had no passage for thirty-six hours. Every means having failed, Hermans insufflated the intestines through the rectum with a bicycle pump attached to the nozzle of a rectal syringe. When the insufflation was considered sufficient the foal was made to rise and to walk. An hour afterward defecation commenced, the colic ceased, and the foal suckled. —*Ann. de Med. Vet.*

Cancer of the Oviduct, Liver, and Kidneys in the Hen. The oviduct was the seat of a tumor as large as an apple, which enclosed a well-formed egg, covered with its shell. The egg could not be expelled on account of the occlusion of the duct. Small, white nodules were disseminated through the tissue of the kidneys. The liver presented a voluminous tumor, 10 cm. long, 8 cm. wide, and 4 cm. thick.

Histological examination of sections of the tumors proved them to be cancer.—*Rec. de Med. Vet.*

